# Applying the Minimal Detectable Change of a Static and Dynamic Balance Test Using a Portable Stabilometric Platform to Individually Assess Patients with Balance Disorders

**DOI:** 10.3390/healthcare8040402

**Published:** 2020-10-14

**Authors:** Juan De la Torre, Javier Marin, Marco Polo, José J. Marín

**Affiliations:** 1 IDERGO-Research and Development in Ergonomics, Biomechanical Laboratory, I3A-University Institute of Research of Engineering of Aragon, University of Zaragoza, 50009 Zaragoza, Spain; 647473@unizar.es (J.M.); jjmarin@unizar.es (J.J.M.); 2 Physical Medicine and Rehabilitation, Hospital of Alcañiz, 44600 Teruel, Spain; marcopoloroyo@gmail.com; 3 Department of Design and Manufacturing Engineering, University of Zaragoza, 50009 Zaragoza, Spain

**Keywords:** posturography, vertigo, vestibular disorders, test-retest, patient-level analysis

## Abstract

Balance disorders have a high prevalence among elderly people in developed countries, and falls resulting from balance disorders involve high healthcare costs. Therefore, tools and indicators are necessary to assess the response to treatments. Therefore, the aim of this study is to detect relevant changes through minimal detectable change (MDC) values in patients with balance disorders, specifically with vertigo. A test-retest of a static and dynamic balance test was conducted on 34 healthy young volunteer subjects using a portable stabilometric platform. Afterwards, in order to show the MDC applicability, eight patients diagnosed with balance disorders characterized by vertigo of vestibular origin performed the balance test before and after a treatment, contrasting the results with the assessment by a specialist physician. The balance test consisted of four tasks from the Romberg test for static balance control, assessing dynamic postural balance through the limits of stability (LOS). The results obtained in the test-retest show the reproducibility of the system as being similar to or better than those found in the literature. Regarding the static balance variables with the lowest MDC value, we highlight the average velocity of the center of pressure (COP) in all tasks and the root mean square (RMS), the area, and the mediolateral displacement in soft surface, with eyes closed. In LOS, all COP limits and the average speed of the COP and RMS were highlighted. Of the eight patients assessed, an agreement between the specialist physician and the balance test results exists in six of them, and for two of the patients, the specialist physician reported no progression, whereas the balance test showed worsening. Patients showed changes that exceeded the MDC values, and these changes were correlated with the results reported by the specialist physician. We conclude that (at least for these eight patients) certain variables were sufficiently sensitive to detect changes linked to balance progression. This is intended to improve decision making and individualized patient monitoring.

## 1. Introduction

Balance disorders have a high prevalence among elderly people in developed countries [1]. Connected with the current trend of increasing age of the population [2,3], this has led to a rise in pathologies that affect balance, causing an increased risk of falls in the elderly population. Around 30% of people aged over 65 years, and more than 50% of individuals in health centers or care homes suffer one or more falls per year, and between approximately 20% and 30% of the global population has had or will have a vertiginous episode in their lives [4,5,6,7,8,9,10].

Therefore, it is necessary to have tools and indicators to assess the response to treatments of pathologies that affect the sensory systems involved in balance: visual, vestibular, and proprioceptive [11]. The effects of a given treatment on a patient through the performance of previous and subsequent tests can help in making decisions about adjusting, changing, or stopping the treatment [12].

Stabilometric platforms are useful in assessing balance because they obtain numerous parameters of the centers or pressure (COP) [13,14]. Stabilometric platforms can assess static balance control and dynamic postural balance through different variables and application methods [15,16]. On the one hand, in static balance control methods (such as the Romberg test), subjects must maintain their COP within the support base throughout the assessment period of time. On the other hand, the assessment of dynamic postural balance, which is vital for motor control, involves measuring the limits of stability (LOS), corresponding to the maximum voluntary angle or distance in which an individual can regulate their COP in a given direction without losing balance [17]. Stabilometric platforms can obtain objective information related to balance pathologies in clinical practice to improve the quality of healthcare and the provided treatments [4,18,19,20].

Therefore, a balance test would be beneficial for clinicians by providing them with an objective assessment device; however, the integration of this type of test in clinical practice involves various difficulties and constraints. Although stabilometric platforms are normally used in the lab, their implementation in the clinic is complex due to difficulty of use and manageability, as well as size, which may call into question clinical applicability [21]. The protocol must be validated and adapted for continuous and assiduous use in the clinic [21,22]. Therefore, it is relevant to conduct studies to measure the degree of variability of a balance test to achieve a successful clinical application.

The minimal detectable change (MDC) index represents the variability of the measures of each variable, i.e., the consistency of the variables, which includes different aspects: the variability of the instrument, the inherent variability of the person and their learning (test duration, visual condition, or position of the feet) and the procedures and protocols applied to perform the test. Therefore, if we obtain a change of one variable that is higher than its MDC value, this would be a relevant change, which could be caused by the treatment effect and not by the test variability [23,24].

No balance assessment studies, based on the MDC results of the test-retest, to detect changes in the results associated with balance progression have been conducted for patients with balance disorders. Numerous test-retest studies have been found in the COP measures but have not included patients with balance disorders in the application [4,19,24,25,26,27,28,29,30,31,32].

Therefore, the aim of this study is to detect relevant changes through MDC values in patients with balance disorders, specifically with vertigo. To address this, the following research actions/steps are proposed: (1) to perform a test-retest of a static and dynamic balance test in a sample of healthy subjects identifying the most sensible variables with the lower MDC values; (2) to analyze whether relevant changes are detected in the results of the balance test before and after the application of a treatment in eight patients diagnosed with balance disorders with vertigo of vestibular origin, contrasting the results with the progression observed by a specialist physician.

## 2. Materials and Methods 

### 2.1. Participants and Ethics Statement

A test-retest study, which consists of repeating the test at two different times with a homogeneous sample of participants under the same conditions, of a static and dynamic balance test was conducted on a sample of 34 healthy young volunteer subjects. Participating in this study were 20 males and 14 females, (age 22.89 ± 3.51 years, height 172.51 ± 9.01 cm, weight 67.38 ± 11.82 kg, body fat index 20.07 ± 9.12 %, foot length [33] 25.39 ± 1.81 cm, abdominal perimeter 79.76 ± 9.77 cm).

The calculation for the choice of 34 subjects was based on the research of Bujang et al. [34], an article in which the relationship between the interclass correlation coefficient (ICC), statistical power, and the number of subjects is established. Taking into account the necessity for two measurements to be made per subject, in order to satisfy the requirement for repetition of the test at two different times, to set a statistical power of 80% and to establish a minimum ICC of 0.5, the sample for analysis needed to number at least 22. Therefore, the choice of 34 subjects is considered appropriate.

The inclusion criteria established for participation in the study were (i) no history of neurological, visual, or vestibular alterations, (ii) no record of musculoskeletal or neurological diseases in the last year, and (iii) no history of limb surgery that may affect the patient’s balance.

It has been demonstrated in several studies that the variability of variables related to the COP are higher in young subjects, which has motivated the selection of the sample [4,28].

As shown in Section 2.5, eight patients with balance disorders characterized by vertigo of vestibular origin were also analysed to apply and assess the test-retest results. The patients were analysed before and after treatment to obtain the individual results of their progression.

The choice of eight patients was based on the sample size calculation for quantitative variables of Charan et al. [35]. The formula used was the following:

*N* = (*Z*_1−_*_α_*_/2_)^2^ × SD^2^/*d*^2^ (1)

where *Z*_1−__α/2_ is the standard normal variate with *p*_v_ < 0.05 (error type 1), corresponding to value of 1.96 (in the majority of studies *p*_v_ values are considered significant below 0.05, hence 1.96 is used in the formula); SD is the standard deviation of the variable (standard deviation value can be taken from previously studies); and d is the absolute error or precision (decided by researcher).

The SD has been selected from the study conducted by Balaguer et al. [20], for using variables and tests similar to those used in this study. Specifically, the test in which all the sensory systems are available has been considered (rigid surface with eyes open), selecting one of the most used variables in balance studies [29,36]: the average speed of the COP. The SD value, for this variable and for this test is 6 mm/s; selecting a d value of 4.1 mm/s, and maintaining a *Z*_1−__α__/2_ value of 1.96, we obtained an *N* value of 8.22 in the eight selected patients.

The selected patients met the following inclusion criteria: (i) between 65 and 75 years old and (ii) having suffered a vertiginous episode in the last year. The following were the exclusion criteria: (i) presented acute osteo-muscular pathology in the lower limbs or lumbar spine, which may alter the outcome of the stabilometric platform, (ii) presented any amputation in the lower limbs, (iii) presented oncological pathology or was in active treatment with chemotherapy, radiotherapy, or hormonal therapy, (iv) presented degenerative diseases (which can cause loss of capacity during the assessment period of time), (v) having been influenced by work or family events (or other kind of events) that have detrimentally affected the patient’s mood.

The study was approved by the Government of Aragon’s Human Research Ethics Committee (CEICA) (16 January 2019). Prior to the beginning of the tests, the participants signed a form, consenting to undergo them and indicating that they understood the aim of the study.

### 2.2. Instrumentation

**Figure 1:**
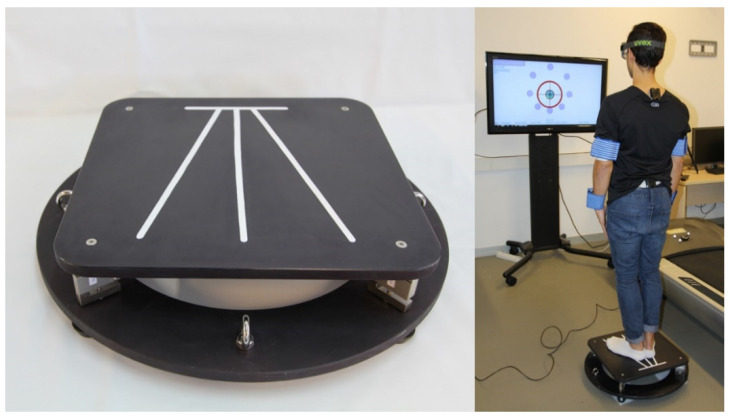
Stabilometric platform and environmental test condition.

The device used was the stabilometric platform MoveHuman-Dyna UZ, which was designed and manufactured by the IDERGO (Research and Development in Ergonomics, University of Zaragoza, Spain) research group (Figure 1). This is a static posturography device designed for research, which comprises four load cells and a lightweight aluminum structure, whose dimensions and characteristics are detailed in the study of [18]. The findings of this device can be replicated in a straightforward manner by other researchers, which enhances the applicability of this study. The acquisition and processing of the platform data, as well as the format and method of exporting them, have been carried out according to the procedure used by [18]. Processing the force data as a function of the cells’ position means we can calculate the real-time position of the trajectory that describes the position of the CoP by applying the appropriate formula [7,13].

Likewise, in accordance with the aforementioned study, the stabilometric platform “meets the standards established by the International Society for Posture and Gait Research (ISPGR) for its clinical application” [37] in relation to various parameters, such as accuracy, precision, linearity, dimensions, resolution, sampling, etc. The precision parameters (accuracy, precision, linearity, dimensions and resolution) were obtained through an experiment in which the metrological characteristics of the platform were tested with a gold standard force platform, along with the error of measurement [17]. Specifically, the gold standard force platform with which it was compared was the AMTI 0R6-7-1000, U.S. Patent Number 4.493.220. The two platforms were positioned one above the other, in order to enable their results to be directly compared and examined for any variability, according to the study of Huurnink et al. (2013) [38]. The stabilometric platform has since been used in several research projects with patients in different hospitals, both public and private; all these research projects have been approved by the CEICA Committee.

The use of this stabilometric platform in this study was justified by its portability characteristics, dimensions, and weight that enable its use by physicians in an examination room, often with limited space [13,18,37,39,40]. A portable device such this platform allows its use in different medical centers due to the quicker installation process and smaller space required, which improves its accessibility and applicability [41,42,43].

### 2.3. Protocol

The static and dynamic balance were both assessed with a set of tests previously applied in other studies [18]. The static balance control was assessed with a test based on the Romberg test and the Modified Clinical Test of Sensory Interaction in Balance (CTSIB-M), with consideration given to four different situations: (1) rigid surface with eyes open (RSEO), (2) rigid surface with eyes closed (RSEC), (3) soft surface with eyes open (SSEO), and (4) soft surface with eyes closed (SSEC). 

On the other hand, the dynamic postural balance was assessed measuring the LOS that a subject is able to reach and, with it, the management capacity of COP [17]. The dynamic LOS test was based on protocols found in the literature [44]. The inclusion of the LOS, complementary to the assessment of the static balance control, provides additional value to the balance assessment protocol [4,8,45]

The protocol applied in the tests (the position of the body, arms and feet during the test [18], environmental conditions (e.g., noise, space, etc.) and the additional instrumentation used as a foam rubber for soft surface, instruments for anthropometric data collection, etc.) is that used by Delatorre et al (2017) for this stabilometric platform (Figure 1). This protocol fulfils certain clinical conditions [21,46,47,48]; it must be fast and should not require multiple repetitions to issue a definite, consistent result [21]. It must also be effective, with clear, specific stages defined by the instructions given by the operator that are understandable by the patient [28].

The test-retest in the sample of healthy subjects was performed on the same day with an interval of 6 hours between the two trials. In this interval, the participants did not perform physical activities that required extra muscular activation and/or caused fatigue that could affect the results of the second trial. The tests of all study participants were coordinated by the same operator. The time interval between the tests performed by the patients was 3 months (process detailed in Section 2.5). 

### 2.4. Statistical Analysis: Test-Retest Study

#### 2.4.1. Selection of Variables

The variables selected for the present test-retest study were those determined by [18] to be more significant in balance assessment studies, whose details and methods are also explained in the same study. The variables selected for the assessment of static and dynamic balance were the range of displacement in the anteroposterior and mediolateral directions, area (surface area covered by the trajectory of the COP), average speed of the COP, and root mean square (RMS) position. Additionally, in the LOS test, two more variables were assessed: the COP limits (maximum displacement reached along each axis of the octagon radii), and the “success” variable (quantification of the management and coordination of the COP along each axis of the octagon radii), both defined in a previous study [18]. 

#### 2.4.2. Minimal Detectable Change Calculation

The MDC index represents the variability of the measures of each variable. If a change of one variable that is lower than its MDC value is detected, it would not be considered relevant, since it is lower than the variability of the test. Therefore, to narrow the intrinsic variability of the test, we considered that the best choice would be to perform the test-retest with healthy and young people (this topic will be justified in the discussion section).

The MDC index was calculated for each of the variables resulting from the test-retest study. The value of the MDC was calculated from the following Equations (2) and (3) [27,30,49,50,51,52,53]:

MDC95% = 1.96 × √2 SEM(2)

SEM = SD-pooled × √(1 − r)(3)

where r is the interclass correlation coefficient (ICC), SD-pooled is the pooled average of the standard deviation of the test and retest, and the SEM is the standard deviation error of measurement.

The dimensionless value of the effect size (MDC.es 95%) was also calculated using Equation (4), which indicates the number of standard deviations that the experiment is capable of detecting [54]:

MDC.es 95% = MDC95%/SD test(4)

where SD test is the standard deviation of the test (initial test).

The ICC results can be classified according to Cicchetti (1994) [55], who provided the following intervals to characterize the ICC inter-rater agreement measures: below 0.40: poor; between 0.40 and 0.59: fair; between 0.60 and 0.74: good; between 0.75 and 1.00: excellent.

### 2.5. Clinical Application Foundations: Assessment of Patients’ Progression

To verify whether the variables in the balance test could detect changes that exceed the MDC index before and after treatment, eight patients diagnosed with balance disorders characterized by vertigo of vestibular origin performed the aforementioned test. This allowed us to verify whether the analyses of the results of the pre-tests and post-tests coincided with the subjective patient progression (positive, null or negative progression) perceived by a specialist physician.

#### 2.5.1. Patients’ Initial Diagnosis

The patients were referred by the Otorhinolaryngology Service of the Alcañiz Hospital (Teruel, Spain) after being diagnosed with a balance disorder. The methods for determining the vestibular deficit were medical history, magnetic resonance imaging, videonystagmography, and tests such as the Dix-Hallpike maneuver (Table 1). 

#### 2.5.2. Clinician 1 Assessment: History and Physical Examination

A doctor (clinician 1) from the Physical Medicine and Rehabilitation Department of the Alcañiz Hospital (Teruel, Spain) evaluated the patients using medical history and a physical examination, as well as functional balance assessment tests such as the Unterberger test [56,57], the up and go test [58,59], and unipodal support test [60]. Clinician 1 prescribed the rehabilitation treatment, which consists of a set of vestibular rehabilitation exercises (to be performed by the patients), which is commonly used in the clinic, establishing these over a period of three months [61,62]. Clinician 1 evaluated the patients at two different times, once before starting the treatment (pre-data), and once three months after starting the treatment (post-data).

#### 2.5.3. Clinician 2 Assessment: Patient Progression Evaluation

The pre- and post- data collected by clinician 1 were assessed by a specialist physician (clinician 2), which allowed an assessment of the balance progression of each of the eight patients. To avoid the results being influenced or contaminated by the interaction between the clinicians, there was no contact between them during the research. The assessment of clinician 2 established three possible categories to evaluate patient progression: positive, null or negative progression.

#### 2.5.4. Magnitude-Based Decision (MBD) to Monitor Patients with Balance Disorders

To measure the effects of a treatment on a specific patient, it is necessary to evaluate the treatment status at different times. Thus, the patient-level approach proposed by Hopkings (2017) [63] was applied to obtain personalized results for each patient. This approach allows the assessment the change between two measurements in an individual through the magnitude-based decision (MBD) method (formerly known as magnitude-based inferences) [64]. 

Hopkings (2017) [63] introduced the patient-level approach providing “A Spreadsheet for Monitoring an Individual’s Changes”. Based on this spreadsheet, we developed a script that applies the MBD method using as input the measurements taken from the pre- and post-balance tests of one specific patient, and the threshold MDC95% previously calculated in the test-retest study. The script was developed using WorldViz-Vizard 6.2 (based on Python 2.7), and the Pandas and Matplotlib libraries. 

The patients in the study performed the Romberg and LOS tests in the rehabilitation clinic of the Alcañiz Hospital on two occasions, once before starting the treatment and another three months after starting the treatment. Using the script that we have developed, it is possible to measure how much each variable has changed and whether the change detected is significant. According to the MBD method, inputs are required to analyze each variable:

- Xdif: difference between the measures taken at two temporal points: pre-value and post-value (Equation (5)). In this case, the pre-value is the measure of each variable just before starting the treatment; and the post-value is the measure three months after starting the treatment.
  (5) $$Xdif=X_{post}-X_{pre}$$
- MBD threshold: for this method, a threshold (numerical value) must be defined from which a change is considered relevant. In our case, we selected the MDC previously calculated.

In this approach, the statistical analysis detects whether the changes exceed a particular threshold, in our case the MDC value. Specifically, we determined where the confidence interval of the difference was located (between pre- and post-tests) in relation to the thresholds of the MDC [54,65]. The intervals of the differences of each variable between the pre-test and post-test results were determined to be on the negative side of the MDC threshold (% negative differences), within the threshold (% trivial differences), or on the positive side (% positive differences), respectively [63]. We deal with the selection of the MDC as threshold in the discussion section.

Firstly, the value and sign (positive or negative) of Xdif is obtained through the difference between the pre-value and post-value. Subsequently, following the calculation method set forth by [63], the probability of change is obtained, which can be defined as the probability that the difference between the two values is relevant. This probability corresponds to the percentage of the confidence interval of the difference (calculated using the Xdif) that is outside of the range (+MDC, −MDC). Finally, the probability that the change is relevant was qualitatively classified (as proposed by Batterham and Hopkins (2006)) [54]. The qualitative classification of the significance of the changes is: most unlikely (<1%), very unlikely (1% to 5%), unlikely (5% to 25%), possibly (25% to 75%), probably (75% to 95%), very likely (95% to 99%) and most likely (>99%).

For the calculations and different graphs presented in this study, Python 2.7 and the numpy, scipy and pandas modules were used.

## 3. Results

### 3.1. Test-Retest Results from Balance Analysis. Minimal Detectable Changes Index

Table 2 shows the results of the test-retest of the variables selected. The values of the means (μ) and standard deviations (SD) of each of the analyzed variables are shown by task. Likewise, the results of the variability through ICC_3,k_ (similar to ICC_2,1_) [24], absolute value of the MDC (95%), and dimensionless value of the effect size (MDC.es 95%) are included.

### 3.2. Results of the Patients-Level Study

**Figure 2:**
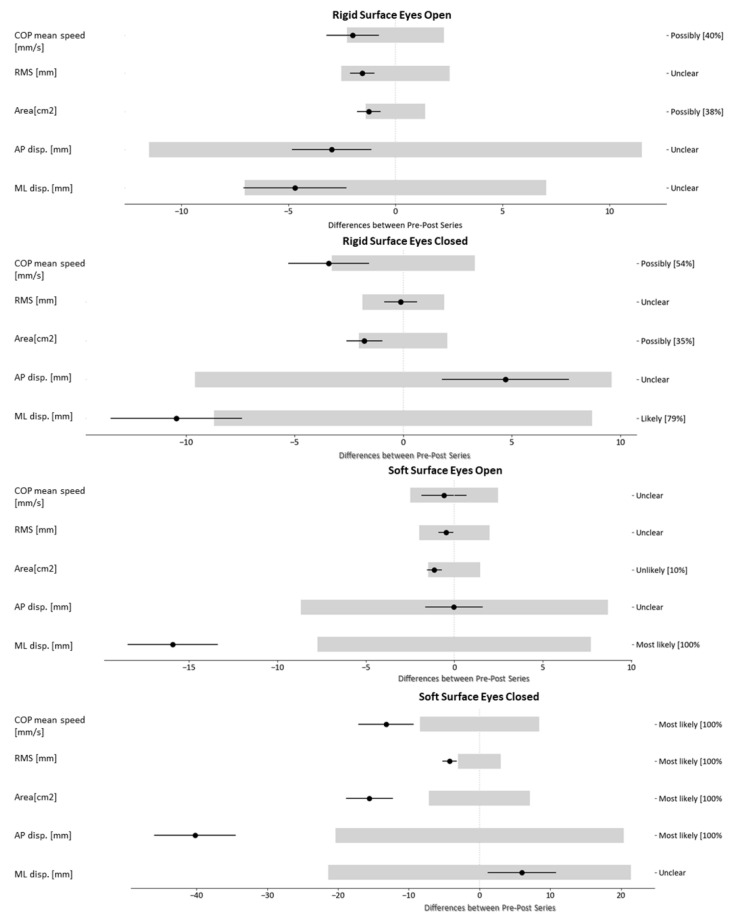
Patient level analysis. Romberg Test. Minimal detectable change (MDC): light grey bars; Differences: thin Black bars.

**Figure 3:**
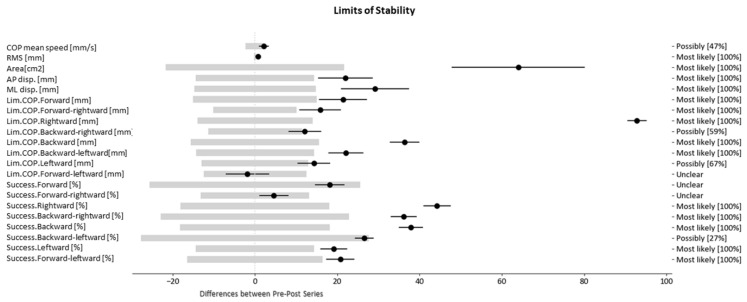
Patient level analysis. Limits of Stability. Minimal detectable change: light grey bars; Differences: Black thin bars.

Due to the large amount of information, the results of the patient with Code 01 are presented in this section as an example, and the results of the remaining patients are presented in the Supplementary Material. Thus, for each of the eight patients, the same information as shown in Table 3 for Patient 1 has been calculated, which includes the values of the (a) variables for the pre- and post-tests, (b) differences, (c) MDC, and (d) percentages of negative, trivial, and positive differences (-/0/+). In addition, Figure 2 and Figure 3 show the produced changes (black lines) in relation to the MDC (light grey rectangle) in each variable for each of the tests.

As shown in Figure 2 and Figure 3, the grey thresholds represent the MDC—or, what is the same—and the change in a variable must exceed the value of this interval in order for us to consider that the change is relevant. So as to quantify each change, the probability that it is relevant is shown in the right margin beside each of the variables.

Analyzing patient 01, most static variables decreased, highlighting the positive or negative differences according to the tests: RSEO: −2 mm/s of COP mean speed; RSEC: −3.4 mm/s of COP mean speed, −10.4 mm of ML displacement; SSEO: −15.9 mm of ML displacement; SSEC: −8.4 of COP mean speed, −4.2 mm in RMS, −15.6 cm^2^ in area and −40.2 mm in AP displacement. In contrast, most dynamic variables increased: +15 mm in Lim.COP.Forward, +15.6 mm in Lim.COP.Backward, +14.3 mm in Lim.COP.Backward-leftward, +25.6% in Success.Forward, and +22.9% in Success.Backward-rightward.

According to the assessment of the specialist physician prior to and after three months from the administration of the treatment, it was observed that the associated clinical symptomatology decreased, and no vertiginous episodes occurred. The physician performed the following functional tests to assess the patient’s balance before and after the treatment was applied: test up and go (15″–12″), Unterberger test (from positive to negative), and unipodal standing time on the right leg (4″–7″) and left leg (3″–10″). Objective improvement in all performed functional tests was demonstrated. Finally, the patient reported improvement in the anamnesis post-treatment, affirming progress in the performance of daily life activities, such as getting out of bed, walking without help, and showering.

The assessment by the specialist physician (positive progression of the patient) and the objective results of the pre- and post-tests (decreased static variables and increased dynamic variables, indicating improvement) coincide.

In relation to the remaining patients, as seen in the Supplementary Material, the physician evaluated two other patients (Codes 02 and 03) with positive progression who experienced objective improvement in the balance test variables. The physician assessed two patients (05 and 06) with negative progression who exhibited objective worsening in the balance test variables. The physician evaluated one patient (04) with no progression without a change in the balance test variables. In addition, the physician assessed another two patients (07 and 08) with no progression who obtained an objective worsening in certain balance test variables. In summary, of the eight patients, an agreement between the physician and the balance test results exists in six, and for two the physician reported no progression, whereas the balance test showed worsening in certain variables.

## 4. Discussion

In this study, a test-retest of the static and dynamic balance test was conducted on a sample of healthy young subjects to identify variables with a lower MDC value. Likewise, eight patients diagnosed with balance disorders characterized by vertigo of vestibular origin performed the test before and after treatment to verify whether the comparative analyses, pre- and post-tests, corroborated the subjective progression perceived by a specialist physician.

The test-retest results are satisfactory in comparison to similar studies found in the literature; specifically, they are improved compared to most of the studies reviewed by [24]. We highlighted the ICC values, which exceeded 0.7, of the COP mean speed and area of the four tasks of static balance, agreeing with the most recent studies in the review (we compare ICC values because they are the most used metrics in the studies consulted). Likewise, taking the study by Salvati et al. (2009) [66] as reference because it is the most recent and is similar to ours, our study shows improvement of the ICC of the area on the soft surface both with eyes open (0.66 vs. 0.33) and closed (0.77 vs. 0.64). Regarding the LOS results, we highlight the COP mean speed and RMS, which slightly improve the ICC values obtained by [21]. Likewise, comparing the ICC values in COP limits and success, greater values are obtained than reported by [25].

The usefulness of a test depends on the MDC value of the variables highlighting in our study the variables with lower MDC in Table 2 (marked with an *). Thus, the lower the MDC of the variables, the more useful the test will be and the greater capacity it will have to detect relevant changes. So the variability of the test is shown, and it is possible to detect variations in balance between different instances of time. 

The presented results and the analysis of the patients have advantages of application; however, certain questions and decisions must be discussed, which are set out below.

The MDC prevents us from mistaking changes due to the ‘noise’ inherent in repeating a test over time. We understand that the existence of ‘noise’ is inseparable from this type of test because the active involvement of the patients is required. 

It is necessary to justify and discuss the motivations and methodological approach chosen in this research. The motivation for the study was to determine the MDC in order to subsequently apply it to patients with balance disorders characterised by vertigo of vestibular origin. Deciding on the set-up for our experimentation, as has been described for the calculation of the MDC, was a matter that led discussions to arise among the multidisciplinary team that participated in this study. 

In the design of the MDC calculation experimentation, it was prioritised that this should aim at studying—and quantitatively limiting—the variation (variability) between the results of pre- and post-testing, specifically, only from those factors intrinsic to the protocol applied and the instrumentation used. The intention was for these factors to be isolated from the noise associated with other factors such as the pathology, the treatment applied between pre- and post-testing, the disease progression, etc. Thus, a sample of healthy and young subjects was selected, since they would enable to study to determine the variability caused by factors such as the intrinsic variability of human beings, the conditions of the tests (body and foot position, duration of the test, visual condition and proprioception, etc.), the learning factor, normal incidents during the course of the tests, or the participant’s own understanding of how to perform the test. Furthermore, it was considered that choosing a sample of healthy subjects would be beneficial for other similar medical tests/research, because in general the recruitment of healthy people is more reliable than accessing a homogeneous sample of patients with a specific pathology.

Likewise, in the experimentation, a period of six hours was established between the two tests for the calculation of the MDC, in order to exclude variability relating to time and, instead, focus on limiting the variables to the factors detailed above. Six hours was also intended to limit variability relating to certain physical activities that healthy subjects could perform between test and retest, (training for weight loss, to gain muscle mass, preparation for an important sporting event, etc.). The six-hour period was also considered to be pertinent as any fatigue factor that could have existed between two closely followed tests was eliminated, as well as the learning factor. The subjects were also controlled in terms of food and drink intake that could affect the test (stimulant drinks, specific diets, etc.), as well as physical activities.

If we would have obtained an MDC from a group of patients with the same pathology and similar age to the presented patients, the influences of the specific treatment could be assessed and that is of interest. However, this restricts the MDC application framework to these patients and does not allow the assessment of changes beyond these patients due to the studied treatment.

According to the above, any change detected between pre- and post-testing in a patient, which exceeds the threshold of the MDC value, is known with certainty not to have been caused by the intrinsic factors already mentioned. Among the extrinsic factors of the test that may affect pre- and post-testing, the effect of the treatment and/or fluctuations of the pathology should be assessed by the clinician in order to analyse the results of the proposed method. However, it must be considered that other factors may also have affected the patient during the assessment period and blur the effects of the treatment; specifically, other diseases that may affect balance (e.g., a degenerative disease) or even personal events (e.g., the death of family members, physical accidents, etc.) that could detrimentally affect the patient’s mood. Regarding this point, we proposed that these considerations should be included in the exclusion criteria included in this study, which could be used in studies of similar theme, in order to limit the extrinsic factors to be assessed from those relative to the treatment administered by the clinician and/or to the fluctuations of the pathology. The first three exclusion criteria were based on [67,68,69,70]; the criteria regarding the presentation of degenerative diseases was based on [71]; and that concerning the influence of personal events was based on [72,73,74]. Therefore, the clinician receives useful information since he can assess the change detected in a patient, excluding the intrinsic factors of the test, allowing him to clearly discern the progression that the patient has followed. This may be relevant to the clinician, who understands the situation of each patient and can inquire about the reasons for these changes.

It is of interest to note that, although a relevant change in a specific variable is obtained in a patient (high probability of change), it is the clinician who must assess whether this change is significant for the pathology that is being evaluated. This is important since, although this type of metric is intended to provide objective information to support a diagnosis, the MDC itself is not a direct diagnosis and should be used only as a reference. If the change detected for a variable is located “outside” the grey area that represents the MDC, it is the clinician who must assess whether this change represents a positive or negative progression, which may be the result of the treatment administered.

Following this line of argumentation, parallel to the study of the MDC for each variable, it would be of interest to inquire about the concept of the minimal important difference [52] (MID) applied in this field. The MID study involves a complex qualitative interpretation process, although its complexity should not detract from its development because the MID index should complement MDC values in the future. In this sense, [75] argued that both values are related, and if it is possible to define a MID value for a specific test, the experiment related to that test must have sufficient accuracy, which is defined by the value of the MDC. Thus, the MID value should preferably be applied, unless the value is lower than that of the MDC, which limits the accuracy of the system.

On the other hand, the use of the MoveHuman-Dyna UZ stabilometric platform does not intend to establish a direct relationship between the findings of the research and the instrumentation used, since it is a platform that can be replicated with easily available materials and devices (e.g., two aluminium plates and four load cells). It is considered that the results obtained and the method followed are easily replicable and can be extrapolated by other researchers; these results are intended to transcend the instrumentation used. The platform is piquing the interest of clinicians as its practical application in the clinic begins to be demonstrated. In relation to this point, it is considered that the platform used provides solutions to the problems of manageability and required space (recurrent in the use of stabilometric platforms), since it is a device with portable characteristics in terms of the little space it requires, its ease of use, and its simple installation.

Regarding the results presented in this study, we highlight the graphs shown in Figure 2 and Figure 3. These graphs were designed to visually and intuitively show the changes detected in the set of variables, thus facilitating the clinician’s quick identification of those variables that require special attention and analysis. Likewise, the opinions of the clinicians, collected in the follow-up meetings of the study, can be summarized in the following sentences: “The numerical results may not be very easy to understand, but with the graphs it is easier identify whether there has been a positive or negative change…”; “The use of this type of graph can help us when we are doubting whether to maintain or change treatment …”.

In the comparative analysis of the results of the patient with Code 01, the pre- and post-treatment balance tests demonstrated the positive progression of the patient, motivated by a decrease in numerous static variables and an increase in dynamics, possibly derived from recovery in the vestibular system of the patient. This improvement coincides with the evaluation of the positive progression of the patient by clinician 2.

Likewise, the balance test results and the specialist physician evaluations were compared for all patients (see Supplementary Material). Of the eight evaluated patients, agreement occurs between those established by clinician 2 and the balance test results in six out of eight cases. In the two mismatches (Patients 07 and 08), the specialist did not establish any changes in the evaluation of the patient, whereas the balance test detected changes that caused worsening in the balance (motivated by a latent process not detected by the doctor). Therefore, as future work, the sensitivity of the balance test should be deepened by analyzing the causes of the discrepancies between the results from the specialist physician and the balance test.

The sample size for comparison of the results of the balance test with the assessment of clinician 2 is perhaps not large. The goal is to exemplify the application of the results at the individual patient level; therefore, one patient would have been enough. However, a sample size of eight patients was obtained through a calculation based on the formula of Charan et al. [35], taking the research of Balaguer et al. [20] as reference; thus illustrating different cases (improvement, worsening, or maintenance).

The variables of each patient were individually analysed in an effort to discern which showed a greater relevant change in relation to the evaluation of clinician 2. On the one hand, among the variables related to the control of static balance, we highlight the following for each of the tests: RSEO, i.e., COP mean speed and ML displacement; RSEC, i.e., COP mean speed, AP displacement, and ML displacement; SSEO, i.e., ML displacement; and SSEC, i.e., COP mean speed, RMS, and area. On the other hand, the variables that presented the greatest relevant change for dynamic postural balance were area, AP displacement and ML displacement, Lim. COP Forward-rightward, Lim. COP Leftward, Success Rightward, Success Leftward, and Success Forward-rightward. Considering the statistical application of the MBD method in the sample of eight patients, these are the variables that are most reliable for assessing change at the individual level and, therefore, for assessing the efficacy of an intervention/treatment. The results have been compared with the study by Tsukamoto et al. (2015) [70] of patients with vestibular complaints, and the following variables coincide as those in which the greatest change was detected: RSEO, i.e., ML displacement; RSEC, i.e., COP mean speed; and SSEC, i.e., COP mean speed. The application of different tests, and the coincidence of only some variables in both studies, implies that there is no greater coincidence between the results.

Derived from the analysis of the patients’ results with agreement from the specialist physician, certain ‘logical’ guidelines of interest were extracted to assess the degree of change as positive or negative, when interpreting the results of individual studies such as this one. A reduction in the variables related to the static balance tasks was associated with an improvement in balance, allowing us to discern whether the improvement comes from the visual, vestibular, or proprioceptive system, depending on in which task the improvement occurs. An increase in the LOS variables reflects an improvement in the dynamic balance and greater control of the COP.

Personalized medicine is a recurrent goal for health professionals [76]. Individual patient assessment, such as that proposed in this study, objectively characterizes the response to treatments such as balance disorders. The conclusions and assessments at the individual level can help recommend each patient to continue, change, or adjust the treatment. The conclusions of the study at the individual level are useful in the clinic, facilitate improved decision-making, and show the benefits of the results.

Regarding the limitations of the study, we consider that the proposed ‘logical’ guidelines can serve as the basis for future studies with patients for the evaluation of treatments; however, it is necessary to deepen the development of rules that allow more efficient qualification of the change detected in the variables, providing the method with greater intelligence for the evaluation of treatments. Likewise, a simple scoring system could be developed (where positive values mean improvement and negative values mean patient deterioration), adding it this to the information already provided by the proposed graphics. On the other hand, we consider that the comparison between the results of the method and the specialist physician assessment should be deepened, establishing a more detailed protocol to more efficiently contrast both evaluations.

Likewise, we assume that the sample of young and healthy subjects could be widened in order to cover other specific age ranges, although we consider it to be adequate for the purpose of this study. On the other hand, future research should include a greater number of patients with different origins of the balance disorder, in order to assess the method’s effectiveness with other kind of pathologies. Likewise, future research should focus on including the MID in conjunction with the MDC; in this way, by determining the MID thresholds in combination with the MDC, research would provide a more effective method with which to assess treatments and support diagnosis. 

## 5. Conclusions

A test-retest of the balance test was conducted on a sample of healthy young subjects, identifying variables that are more reproducible with a lower MDC value: the COP mean speed in the RSEO, RSEC, SSEO tasks, the LOS, RMS, and area in RSEC and LOS, and all the COP limits in the LOS. Regarding the assessment of the eight patients with balance disorders characterized by vertigo of vestibular origin, the evaluations reported by the specialist physician mostly coincide with the objective pre- and post-test results. In this way, the applicability of the results to the assessed patient was shown because certain variables were sufficiently sensitive to detect important changes linked to a progression (improvement/worsening/no changes) in balance. The results of this study aim is to improve medical decision-making and the individualized follow-up of patients.

## Figures and Tables

**Table 1:** Data of patients assessed.

Patient Code	Age	Gender	Anamnesis and Examination
01	72	Male	BPPV with associated signs of bilateral hearing loss (detected by audiometry) and nystagmus.
02	73	Female	Ménière syndrome.
03	70	Male	BPPV with associated signs of bilateral hearing loss (detected by audiometry).
04	73	Female	Osteosclerosis. Bilateral hearing loss.
05	68	Male	Ménière syndrome. Unilateral tinnitus and pathological nystagmus.
06	74	Male	Ménière syndrome. Unilateral tinnitus.
07	68	Female	Vestibular hypofunction. Pathological nystagmus.
08	75	Male	BPPV with associated signs of bilateral hearing loss (detected by audiometry).

BPPV: Benign Paroxysmal Peripheral Vertigo.

**Table 2:** Test-retest results from balance tests. Minimal detectable changes index.

Balance Tasks	Variables	Test µ (SD)	Retest µ (SD)	ICC	MDC95_es	MDC95
RSEO	COP mean speed [mm/s] *	8.1 (2.3)	8.4 (2.2)	0.87	0.9	2.3
	RMS [mm]	5.1 (1.2)	5.3 (1.7)	0.61	2.1	2.6
	Area [cm^2^]	1.7 (0.9)	1.7 (0.9)	0.71	1.5	1.4
	AP disp. [mm]	19 (4.7)	19.5 (6.1)	0.42	2.4	11.6
	ML disp. [mm]	15.8 (4.4)	15.8 (4.8)	0.70	1.5	7.1
RSEC	COP mean speed [mm/s] *	13.9 (4.2)	13.6 (3.9)	0.92	0.7	3.3
	RMS [mm] *	6.2 (1.9)	6.5 (1.8)	0.87	0.9	1.9
	Area [cm^2^] *	3.5 (1.9)	3.4 (1.8)	0.85	1.0	2.0
	AP disp. [mm]	24.3 (6.1)	25.9 (7.7)	0.75	1.5	9.7
	ML disp. [mm] *	22.4 (8.3)	22.7 (7.6)	0.85	1.0	8.7
SSEO	COP mean speed [mm/s] *	13.7 (2.7)	13.5 (2.3)	0.88	0.9	2.5
	RMS [mm]	6.9 (1.3)	6.9 (1.5)	0.74	1.4	2.0
	Area [cm^2^]	3.7 (1.0)	3.4 (1.1)	0.76	0.4	1.5
	AP disp. [mm]	27.5 (5.1)	27.2 (6.7)	0.72	1.7	8.8
	ML disp. [mm]	23.4 (4.3)	22.2 (4)	0.56	1.7	7.7
SSEC	COP mean speed [mm/s] *	36.2 (7.9)	34 (6.6)	0.83	1.0	8.5
	RMS [mm]	13.6 (2.2)	13.3 (2.3)	0.77	1.3	3.0
	Area [cm^2^]	17.2 (5.3)	15.7 (4.7)	0.74	1.3	7.2
	AP disp. mm]	54.8 (12.4)	52.7 (10.9)	0.61	1.6	20.4
	ML disp. [mm]	51.6 (10.6)	50.4 (9.5)	0.42	2.0	21.4
LOS	COP mean speed [mm/s] *	15.9 (3.1)	15.4 (3.0)	0.93	0.7	2.3
	RMS [mm] *	5.8 (0.3)	5.9 (0.3)	0.96	0.5	0.2
	Area [cm^2^] *	179.5 (43.7)	183.2 (45.2)	0.97	0.4	21.7
	AP disp. [mm]	153.8 (20.4)	157 (19.7)	0.94	0.7	14.4
	ML disp. [mm]	157.1 (20)	158 (23.1)	0.94	0.7	14.8
	Lim.COP.Forward [mm] *	84.6 (17.5)	87.5 (15.3)	0.9	0.8	15.0
	Lim.COP.Forward-rightward [mm] *	90.4 (14.4)	91.8 (11.8)	0.93	0.7	10.2
	Lim.COP.Rightward [mm]	77.3 (12.2)	75.5 (12.2)	0.83	1.1	14.0
	Lim.COP.Backward-rightward [mm]	68.2 (12.7)	68 (10.6)	0.88	0.8	11.4
	Lim.COP.Backward [mm]	68.1 (14.3)	68.5 (13.6)	0.84	1.0	15.6
	Lim.COP.Backward-leftward [mm]	71.2 (12.2)	71 (14.1)	0.85	1.1	14.4
	Lim.COP.Leftward [mm]	77.6 (10.5)	81.4 (13.8)	0.86	1.2	13.1
	Lim.COP.Forward-leftward [mm] *	91 (14.8)	92.6 (12.4)	0.89	0.8	12.5
	Success.Forward [%]	75.7 (14.4)	76.8 (11.7)	0.5	1.7	25.8
	Success.Forward-rightward [%]	79.4 (12.2)	79.1 (13.9)	0.87	1.0	13.2
	Success.Rightward [%]	78.3 (12.3)	80.4 (10.7)	0.68	1.4	18.1
	Success.Backward-rightward [%]	79.1 (11.6)	78 (9.9)	0.42	1.9	23.0
	Success.Backward [%]	83.5 (11.)	85.6 (8.2)	0.57	1.5	18.5
	Success.Backward-leftward [%]	80.4 (10.5)	82.8 (11.7)	0.2	2.6	27.7
	Success.Leftward [%]	80.8 (11.7)	81.6 (11.1)	0.8	1.2	14.4
	Success.Forward-leftward [%]	78.8 (13.3)	80.5 (11.4)	0.77	1.2	16.5

µ: mean; SD: standard deviation; ICC: intraclass correlation coefficient; MDC95_es: minimal detectable change in dimensionless value effect size at 95%.; MDC95: minimal detectable change in absolute value at 95%; RSEO: rigid surface, open eyes; RSEC: rigid surface, eyes close; SSEO: soft surface, eyes open; SSEC: soft surface, eyes close; LOS: limits of stability; COP: center of pressure; RMS: root mean square; AP: anteroposterior; ML: mediolateral. * Balance variables selected with higher ICC.

**Table 3:** Results of the patient 01.

Balance Tasks	Variables	Value Pre	Value Post	Difference	MDC	% (− / 0 / +)
RSEO	COP mean speed [mm/s]	13.3	11.2	−2.0	2.3	41/59/0
	RMS [mm]	6.5	5.0	−1.5	2.6	22/78/0
	Area [cm^2^]	3.4	2.1	−1.2	1.4	43/57/0
	AP disp. [mm]	20.0	17.0	−3.0	11.6	8/91/1
	ML disp. [mm]	22.4	17.7	−4.7	7.1	26/74/0
RSEC	COP mean speed [mm/s]	20.3	16.9	−3.4	3.3	53/47/0
	RMS [mm]	7.6	7.5	−0.1	1.9	4/94/2
	Area [cm^2^]	5.1	3.3	−1.8	2.0	40/60/0
	AP disp. [mm]	26.9	31.6	4.7	9.7	0/84/16
	ML disp. [mm]	30.4	20.0	−10.4	8.7	65/35/0
SSEO	COP mean speed [mm/s]	18.5	18.0	−0.6	2.5	7/92/1
	RMS [mm]	7.3	6.8	−0.5	2.0	7/92/1
	Area [cm^2^]	4.9	3.7	−1.1	1.5	33/67/0
	AP disp. [mm]	23.3	23.3	0.0	8.8	3/94/3
	ML disp. [mm]	44.3	28.4	−15.9	7.7	98/2/0
SSEC	COP mean speed [mm/s]	62.6	49.4	−13.2	8.5	86/14/0
	RMS [mm]	19.2	15.0	−4.2	3.0	78/22/0
	Area [cm^2^]	41.0	25.4	−15.6	7.2	99/1/0
	AP disp. [mm]	102.4	62.2	−40.2	20.4	97/3/0
	ML disp. [mm]	66.5	72.5	6.0	21.4	1/91/8
LOS	COP mean speed [mm/s]	19.0	21.2	2.2	2.3	0/52/48
	RMS [mm]	5.5	6.2	0.7	0.2	0/0/100
	Area [cm^2^]	170.0	234.0	64.0	21.7	0/0/100
	AP disp. [mm]	154.9	176.8	21.9	14.4	0/16/84
	ML disp. [mm]	150.3	179.5	29.2	14.8	0/3/97
	Lim.COP.Forward [mm]	86.8	108.2	21.4	15.0	0/21/79
	Lim.COP.Forward-rightward [mm]	9.5	108.3	15.8	10.2	0/14/86
	Lim.COP.Rightward [mm]	0.0	92.8	92.8	14.0	0/0/100
	Lim.COP.Backward-rightward [mm]	60.4	72.5	12.1	11.4	0/45/55
	Lim.COP.Backward [mm]	32.7	69.0	36.4	15.6	0/1/99
	Lim.COP.Backward-leftward [mm]	60.0	82.1	22.1	14.4	0/15/85
	Lim.COP.Leftward [mm]	72.7	87.0	14.3	13.1	0/43/57
	Lim.COP.Forward-leftward [mm]	106.6	104.7	−1.9	12.5	5/93/2
	Success.Forward [%]	50.7	68.8	18.2	25.8	0/72/28
	Success.Forward-rightward [%]	69.0	73.6	4.6	13.2	1/89/11
	Success.Rightward [%]	43.9	88.2	44.2	18.1	0/0/100
	Success.Backward-rightward [%]	45.3	81.5	36.2	23.0	0/13/87
	Success.Backward [%]	53.9	91.9	37.9	18.5	0/2/98
	Success.Backward-leftward [%]	43.6	70.2	26.6	27.7	0/53/47
	Success.Leftward [%]	55.9	75.1	19.1	14.4	0/26/74
	Success.Forward-leftward [%]	58.5	79.2	20.8	16.5	0/31/69

## Supplementary Materials

The following is available online at https://www.mdpi.com/2227-9032/8/4/402/s1, S1: Supplementary Material. Patient Level Study.
